# Androgen receptor expression in patients with triple negative breast cancer treated with neoadjuvant chemotherapy: a single institution study

**DOI:** 10.7150/jca.67536

**Published:** 2022-05-09

**Authors:** Nithya Sridhar, Chad Glisch, Zeeshan Jawa, Lubna N. Chaudhary, Sailaja Kamaraju, John Burfeind, John Charlson, Christopher R. Chitambar, Julie M. Jorns, Yee Chung Cheng

**Affiliations:** 1 Division of Hematology and Oncology, Department of Medicine, Medical College of Wisconsin, Milwaukee, Wisconsin, USA.; 2 Department of Pathology, Medical College of Wisconsin, Milwaukee, Wisconsin, USA.

## Abstract

**Background:** Androgen receptor (AR) expression has emerged as a potential prognostic and predictive marker in patients with triple negative breast cancer (TNBC). We conducted a retrospective analysis to evaluate pathologic complete response (pCR) rates, disease-free survival (DFS) and overall survival (OS) in patients with AR positive and AR negative TNBC treated with neoadjuvant chemotherapy.

**Methods:** 107 patients with TNBC subtype, treated with neoadjuvant chemotherapy between June 2006 and March 2016 were evaluated for AR expression. Androgen receptors were evaluated by immunohistochemical staining (clone AR441, Dilution 1:50, Dako-Agilent, Santa Clara, CA) using whole tissue sections from archived paraffin-embedded formalin-fixed (FFPE) blocks. AR positive was defined as ≥10% nuclear stained cells. Correlation of AR expression was examined with age, BMI, race, menopausal status, tumor grade, tumor size, and lymph node involvement, and response and outcomes. Univariate and multivariate analyses were performed to determine an association with AR expression and pathologic response and survival outcomes.

**Results:** Fifty-eight patients with available tumor specimens were stained, with twenty (34.5%) being AR-positive and thirty-eight (65.5%) being AR negative. Median age was 49 years and median follow up was 5.7 years. All patients received anthracycline based neoadjuvant chemotherapy with 13 patients (23%) receiving an additional platinum chemotherapy. BRCA mutation positivity was 7% for the entire group. No differences in age, menopausal status, BMI, race, tumor size and lymph node involvement were observed between the two groups. However, there was a statistically significant difference in tumor grade between the two groups (p=0.008). Overall pCR rate was 28% with no difference between the two groups (30% vs 26%, p=0.56). There was no statistically significant difference in median DFS (5.9 years vs 5.2 years (*p*=0.94) and median OS (6.2 years vs 5.4 years, *p*=0.98) between the AR positive and AR negative groups.

**Conclusions:** Our study did not find an association of AR status and the pathologic responses or survival outcomes in patients with TNBC treated with neoadjuvant chemotherapy. Further studies exploring the prognostic and predictive role of AR in patients with TNBC are warranted.

## Introduction

Triple negative breast cancer (TNBC) is a subtype of breast cancer that accounts for 15-20% of all invasive breast cancers 1, 2. TNBC is characterized by the absence of estrogen and progesterone receptor expression, and a lack of overexpression of human epidermal growth factor 2 (HER2). Surgery and radiation are used as local treatment, but the main systemic treatment for patients with TNBC is cytotoxic chemotherapy due to a lack of known identified markers to target. Despite these treatment options, patients with TNBC are shown to have an unfavorable prognosis compared to hormone-sensitive breast cancer phenotypes, with worse breast cancer specific survival and overall survival in the first three years. Patients with TNBC also have higher rates of recurrence, metastasis to distant areas, and an overall more aggressive nature when compared to the other breast cancer subsets 3.

Due to the aggressive biology and fewer options of targeted therapy for TNBC patients, identifying a target aimed treatment that can improve outcomes in these patients is especially important. Androgen receptors (AR) are a class of nuclear steroid receptor that are overexpressed in many major breast cancer phenotypes at an approximate rate of 70-90%. Its' expression in TNBC varies from 10-50% 4-8. The association between AR expression and its prognostic role in TNBC has been an area of debate. Although initial studies have reported that patients with AR-positive TNBC correlated with poorer outcomes 9-11, increasing studies have showed patients with AR-positive TNBC associated with a longer disease-free survival and overall survival compared to patients with AR-negative TNBC 12-15. The expression of AR in TNBC seemed to make the prognosis more favorable like the endocrine sensitive breast cancer subtypes. Another example of an androgen dependent tumor with a favorable prognosis is prostate cancer. The predictive value of AR in tumor response has been clearly observed following AR-directed therapy in prostate cancer, but its predictive role in the treatment of TNBC is still to be determined. Several androgen antagonist treatments have been studied for patients with AR positive TNBC with promising results 16-19. The AR expression has made the TNBC endocrine sensitive. However, the presence of AR expression itself may also predict a lower response rate to the standard cytotoxic chemotherapy similar to the endocrine sensitive breast cancer subtypes. Therefore, unanswered questions about the role of AR in TNBC remain. We hereby retrospectively evaluated the outcomes of patients with AR positive and AR negative TNBC treated with standard neoadjuvant chemotherapy in our institution.

## Materials and Methods

This study identified patients with non-metastatic TNBC at the Froedtert Clinical Cancer Center who were treated with neoadjuvant chemotherapy between June 2006 and March 2016. All the TNBC patients included were eligible for neoadjuvant chemotherapy and received the standard anthracycline and taxane containing regimen. The use of platinum was on the discretion of the treating physician. A retrospective chart review was performed, and data was supplemented from the institution's electronic medical record system. Androgen receptors were evaluated by immunohistochemical staining (clone AR441, Dilution 1:50, Dako-Agilent, Santa Clara, CA) using whole tissue sections from archived paraffin-embedded formalin-fixed (FFPE) blocks. AR positivity was defined by ≥10% nuclear staining of tumor cells 20. Pathologic complete response (pCR) was defined as no residual invasive breast carcinoma or metastatic carcinoma in ipsilateral axillary lymph nodes. Disease-free survival (DFS) was defined as the interval between the date of diagnosis and the date for which relapse was confirmed or the date of the most recent clinic appointment. Overall survival (OS) was defined as the interval between the date of diagnosis and the date of most recent clinic appointment if the patient is living or the date of death. Statistical analysis was performed using Microsoft Excel and data presented were expressed as numerical comparisons. Simple descriptive statistics and chi squared analyses were used to visualize differences. Correlation of AR expression and its association with multiple prognostic factor outcomes including age, BMI, race, menopausal status, tumor grade, tumor size, and lymph node involvement, were analyzed the using chi squared tests. A *p-*value of ≤ 0.05 was considered statistically significant for all of the analyses.

## Results

### Demographics

A total of 107 patients with non-metastatic TNBC were identified in this study. Of these patients, 58 patients had available tumor specimens for AR testing, with 20 (34.5%) being AR-positive and 38 (65.5%) being AR negative. Twenty-five patients were premenopausal and 33 were post-menopausal. Patient race comprised: 41 Caucasian, 16 African American, and 1 Hispanic (Table 1). The median age of diagnosis was 49 years old [range, 29 - 81] and median follow up for this cohort was 5.7 years [range, 0.7 - 14.1].

### Patient and Tumor Characteristics

All 58 patients received standard anthracycline and taxane based neoadjuvant chemotherapy with 13 patients (23%), 6 in AR positive group and 7 in AR negative group, receiving an additional platinum chemotherapy. The studies were conducted before the practice of adjuvant capecitabine. Therefore, no patient received capecitabine. BRCA mutation positivity was 7% for the entire group. The average tumor size for the 58 tumor specimens was 3.8 cm ± 1.8 cm, with 14 patients having a tumor larger than 5 cm. Twenty-eight patients (48.2%) had lymph node involvement before surgery with 10 in AR positive group and 18 in AR negative group (Table 1). Between the two groups of AR expression, there were no differences observed based on age, menopausal status, BMI, race, tumor size, lymph node involvement and the use of platinum. However, there was a difference in tumor grade between the two groups with the AR positive group having a greater percentage of grade 2 tumors (25% vs 2.6%), and a lesser percentage of grade 3 tumors (75% vs 97.4%) when compared to the AR negative group (p=0.008).

### Pathological Complete Response

The overall pCR rate was 28% using standard anthracycline and taxane +/- platinum containing regimens. There was no statistically significant difference between the two groups, with the AR positive group having a pCR rate of 30% and the AR negative group having a pCR rate of 26% (*p*=0.56).

### Disease-free Survival

The median DFS was 5.4 years [range, 0.3 - 12]. The AR positive cohort had a median DFS of 5.9 years [range, 0.8 - 11.3], whereas the AR negative cohort had a median DFS of 5.2 years [range, 0.3 - 12], however this difference was not statistically significant (*p*=0.94).

### Overall Survival

The median OS was 5.7 years [range, 0.7 - 14.1]. The AR positive cohort had a median OS of 6.2 years [range, 1.0 - 11.3], whereas the AR negative cohort had a median OS of 5.4 years [range, 0.7 - 14.1]. There was also no significant difference between the two groups in terms of overall survival (*p*=0.98).

## Discussion

The results of this study did not find an association of AR status and the pCR rate, survival outcomes including DFS and OS, or various clinicopathological factors except the grade, in patients with non-metastatic TNBC who underwent standard neoadjuvant chemotherapy. AR positive tumors had lower grade than AR negative tumors.

Androgen receptor expression has been noted in TNBC, with an expression rate of 10-50% 4-8. The variability in frequency of the AR expression in TNBC is mostly due to the lack of standard assay used for breast cancer and the use of different assay cutoffs. The standard AR assay for prostate cancer is well established, but different anti-AR antibodies have been used in breast cancer. Most studies have used 10% as the cutoff for AR positivity as recommended by the College of American Pathology previously for the estrogen/progesterone receptors in breast cancer before the change of cutoff to 1%. However, Astvatsaturyan et al suggested a 1% cutoff as the appropriate threshold for AR positivity 21. In the preclinical setting, Barton et al have shown that the TNBC cell lines with AR expression as low as 1% could demonstrate a response to androgen antagonist agent like enzalutamide, although higher AR expression levels may be associated with a better response 22. However, several clinical studies did not show that association. Traina et al reported a phase II study of the use of enzalutamide for the treatment of AR expressed TNBC using ≥ 1% as AR positivity cutoff, where the clinical benefit rate (CBR) was 25% and the median progression-free survival (PFS) was 11.6 weeks 17, 18. Two other studies using a higher AR positivity cutoff reported similar results. Gucalp et al reported a CBR of 19% and median PFS of 12 weeks while using bicalutamide in AR positive TNBC 16, and Bonnefoi et al reported a CBR of 20% and a median PFS of 11.2 weeks while using abiraterone in AR positive TNBC 19. Although these clinical studies demonstrated the activity of single agent anti-androgen in AR positive TNBC, the clinical efficacy was limited to be used in clinical practice. Currently no studies have suggested a prognostic or predictive benefit of increasing AR level. In our analysis of 58 TNBC patient specimens, 34.5% showed AR positivity using the ≥10% cut off for positivity. Eight patients (13.8%) demonstrated a high AR level of ≥ 50% with 4 of them showed a very high-level AR of ≥ 80%. However, the sample size was not enough to have meaningful analysis. The fact that a good number of triple-negative tumors displayed AR expression with > 10% at high level warrants additional studies concerning its association with clinical outcomes and possible treatment options.

Androgen receptor expression and positivity is emerging as a possible prognostic indicator of early breast cancer. A meta-analysis performed of over 10,000 patients with early-stage ER positive, ER negative, HER2 positive, or TNBC demonstrated a longer disease-free survival (HR=0.61, p<0.001) and overall survival (HR=0.62, p<0.001) among AR positive patients. Upon breast cancer subtype breakdown, AR positivity showed similar results of improved prognosis in both ER positive and TNBC patients 12. Another meta-analysis of 521 TNBC patients reported by Kim et al also showed the association of AR expression with a benefit in both DFS (OR=0.44, p=0.002) and OS (OR=0.26, p=0.001) 13. However, Wang et al reported the meta-analysis of 2826 TNBC patients with a 24.4% AR positivity. Patients with AR expression tended to have lower tumor grade (p<0.001) but more lymph node involvement (p<0.01). Although AR positivity was associated with prolonged DFS (HR=0.809, p<0.05), no benefit in OS was seen (HR=1.27, p=0.168) 14. Qu et al also reported a meta-analysis of 5270 patients which showed a 65.2% AR positivity with a benefit in DFS in the TNBC subgroup (HR=0.4) but not in OS (HR=1.17) 15. In summary, multiple meta-analyses have demonstrated the association of AR expression with at least DFS. Although our study did not show any benefit in DFS or OS for AR positive TNBC, we showed that the AR expression was associated with lower grade, a numerical trend of decreased lymph node involvement and lower clinical stage although it was not statistically significant.

There has also been growing evidence that AR expression may be a clinical predictor of pCR to chemotherapy. Loibl et al evaluated AR expression and the pCR rate in the phase III GeparTrio trial which showed the pCR rate was 25.4% in patients with AR negative breast cancer but only 12.8% in patients with AR positive breast cancer 23. Masuda et al showed that among the 20 patients with TNBC that were classified as LAR subtype according to molecular gene expression, none had pCR to neoadjuvant chemotherapy 24. Asano et al published that the rate of pCR was significant higher in patients with TNBC (n=61) with AR negative tumor specimens (63.2%) compared to the AR positive cohort (17.4%) with a p value of 0.001 25. Although AR expression was associated with lower response to neoadjuvant chemotherapy, it was associated with improved survival outcomes. This further demonstrated the similar biology of AR positive TNBC and endocrine sensitive breast cancer.

Our study had several limitations. The first was the AR assay method. Like other clinical studies, we used the immunohistochemical stain method to assay the AR protein expression. However, Lehmann et al reported a study of the use of AR antagonist enzalutamide and PI3K inhibitor taselisib in patients with AR positive metastatic TNBC, where the use of molecular profiling to identify the luminal androgen receptor (LAR) subtype may be a better way to identify patients to benefit from antiandrogen therapy 26. Molecular profiling has the advantage of identifying different subtypes of breast cancer and selecting certain subtypes to the targeted treatment. However, it is not readily used for routine clinical practice and its expenditure is another issue. The other limitation was the AR positivity cutoff. Although 1% is the current cut off used to define ER and PR positivity in breast cancer according to the latest ASCO/CAP guidelines 27, our institution used an AR positivity cut-off of 10%, like other studies of this nature. The optimal AR positivity cutoff has yet to be determined. Our results may be impacted by the variability of criteria used to define AR positivity. The last and most important limitation was the small sample size. Although we had identified 107 eligible patients in our study, many of the tumor samples were not available for the AR assay due to various clinical reasons. The small number of 58 patients with available tumor samples did not allow us to have additional data analysis including looking at different AR positivity cutoffs or analyzing the correlation with different AR levels. Therefore, we recommend cautious interpretations of our results. Nevertheless, due to the poor prognosis and lack of targeted therapeutics for TNBC compared to other breast cancer subtypes, there is an imminent need to find effective treatments for this patient cohort. Since anti-androgen therapy is a relatively safe treatment with manageable side effects, AR remains a potential target for breast cancer. Further studies exploring the prognostic and predictive role of AR in patients with TNBC are warranted.

## Figures and Tables

**Table 1:** Demographic variables and tumor characteristics in AR positive and AR negative patients

	AR positive (n=20)	AR negative (n=38)	Total (n=58)	P-value
Race				*0.63*
Caucasian	15 (75.0%)	26 (68.4%)	41 (70.7%)	
Black or African American	4 (20.0%)	12 (31.6%)	16 (27.6%)	
Hispanic	1 (5.0%)	0 (0.0%)	1 (1.7%)	
Menopausal status				*0.73*
Pre-menopausal	8 (40.0%)	17 (44.7%)	25 (43.1%)	
Post-menopausal	12 (60.0%)	21 (55.3%)	33 (56.9%)	
Tumor size				*0.43*
T1	2 (10.0%)	1 (2.6%)	3 (5.2%)	
T2	13 (65.0%)	20 (52.6%)	33 (56.9%)	
T3	4 (20.0%)	13 (34.2%)	17 (29.3%)	
T4	1 (5.0%)	3 (7.9%)	4 (6.9%)	
Unknown	0	1 (2.6%)	1 (1.7%)	
Lymph node involvement				*0.61*
N0	10 (50.0%)	20 (52.6%)	30 (51.7%)	
N1	8 (40.0%)	10 (26.3%)	18 (31.0%)	
N2	1 (5.0%)	3 (7.9%)	4 (6.9%)	
N3	1 (5.0%)	5 (13.2%)	6 (10.3%)	
Grade				0.08*
2	5 (25.0%)	1 (2.6%)	6 (10.3%)	
3	15 (75.0%)	37 (97.4%)	52 (89.7%)	
Stage				*0.62*
I	1 (5.0%)	1 (2.6%)	2 (3.4%)	
II	14 (70.0%)	23 (60.5%)	37 (63.8%)	
III	5 (25.0%)	14 (36.8%)	19 (32.8%)	

* Indicates statistical significance (*p ≤ 0.05*)
